# Macromolecular Cargo Encapsulation via In Vitro Assembly of Two‐Component Protein Nanoparticles

**DOI:** 10.1002/adhm.202303910

**Published:** 2024-02-11

**Authors:** Karla‐Luise Herpoldt, Ciana L. López, Isaac Sappington, Minh N. Pham, Selvi Srinivasan, Jason Netland, Katherine S. Montgomery, Debashish Roy, Alexander N. Prossnitz, Daniel Ellis, Adam J. Wargacki, Marion Pepper, Anthony J. Convertine, Patrick S. Stayton, Neil P. King

**Affiliations:** ^1^ Department of Biochemistry University of Washington Seattle WA 98195 USA; ^2^ Institute for Protein Design University of Washington Seattle WA 98195 USA; ^3^ Department of Bioengineering University of Washington Seattle WA 98195 USA; ^4^ Department of Immunology University of Washington Seattle WA 98195 USA; ^5^ Present address: 2seventy Bio Seattle WA 98102 USA; ^6^ Present address: Department of Material Science and Engineering Missouri University of Science and Technology Rolla MO 65409 USA

**Keywords:** computational protein design, encapsulation, polymer prodrug, protein nanomaterials, RAFT, RNA delivery, self‐assembly, targeted delivery, vaccine adjuvants

## Abstract

Self‐assembling protein nanoparticles are a promising class of materials for targeted drug delivery. Here, the use of a computationally designed, two‐component, icosahedral protein nanoparticle is reported to encapsulate multiple macromolecular cargoes via simple and controlled self‐assembly in vitro. Single‐stranded RNA molecules between 200 and 2500 nucleotides in length are encapsulated and protected from enzymatic degradation for up to a month with length‐dependent decay rates. Immunogenicity studies of nanoparticles packaging synthetic polymers carrying a small‐molecule TLR7/8 agonist show that co‐delivery of antigen and adjuvant results in a more than 20‐fold increase in humoral immune responses while minimizing systemic cytokine secretion associated with free adjuvant. Coupled with the precise control over nanoparticle structure offered by computational design, robust and versatile encapsulation via in vitro assembly opens the door to a new generation of cargo‐loaded protein nanoparticles that can combine the therapeutic effects of multiple drug classes.

## Graphical abstract

The use of a computationally designed, two‐component icosahedral protein nanoparticle is reported to encapsulate multiple macromolecular cargoes via simple and controlled self‐assembly in vitro. In addition, immunogenicity studies of nanoparticles packaging synthetic polymers carrying a small‐molecule TLR7/8 agonist increase humoral immune responses while minimizing systemic cytokine release.

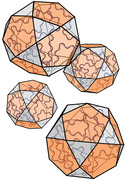

## 1 Introduction

Nanomaterials have emerged as a promising solution to targeted delivery,^[^ ^1^ ^]^ and nanoparticles in particular have several attractive features. For example, their large surface‐to‐volume ratio allows the encapsulation of drug molecules at high density, and, in favorable cases, the ability to engineer the exterior surface independently of the interior volume enables the inclusion of targeting moieties.^[^ ^2^ ^]^ Much of the research in this area has focused on the development of synthetic structures, such as lipid nanoparticles and polymeric micelles. The encapsulation of cargoes within these materials is usually driven by covalent linkage or hydrophobic interactions in an aqueous environment.^[^ ^3^, ^4^, ^5^, ^6^ ^]^ Precise chemical manipulation of their building blocks also gives polymeric nanomaterials several strengths, including modification of drug‐nanoparticle linker composition to tune release rates,^[^ ^7^, ^8^ ^]^ control of colloidal size in vivo using thermal‐ or pH‐responsive monomers,^[^ ^9^, ^10^ ^]^ and selection of biodegradable polymers to minimize the toxicity profile of the drug and its carrier.^[^ ^11^, ^12^ ^]^ Despite these advantages, few such carriers have been licensed for human use. A major limitation to their development is particle heterogeneity, both in terms of size and drug‐loading density, which can substantially impact stability, potency, and targeting.^[^ ^13^, ^14^ ^]^ Furthermore, the chemical simplicity of these molecules fundamentally limits the information, and therefore functionality, that can be encoded in them. Nevertheless, lipid nanoparticles in particular have had an enormous impact on human health recently in the form of mRNA vaccines for SARS‐CoV‐2, highlighting their potential for broader clinical use.^[^ ^15^ ^]^

In contrast to synthetic nanomaterials, there is growing interest in the use of proteins as drug delivery vehicles. Antibody‐drug conjugates (ADCs) exemplify how the exquisite specificity of protein–protein interactions can be used to deliver a toxic molecule to its target cell.^[^ ^16^ ^]^ However, these systems are hindered by the limited number of drug molecules that can be conjugated to each antibody, the stability of the conjugated drug in vivo, and the complex chemistries that are required to ensure the cargo remains bioactive post‐conjugation. Because of these restrictions, most drugs delivered in this way are small molecules, although a few systems have been described whereby antibodies are used to deliver biologics.^[^ ^17^ ^]^

Protein‐based nanoparticles are an emerging alternative to conventional materials that combine the modalities described above. Inspired by the most efficient example of nanoscale targeted delivery—viruses—the use of such nanoparticles offers many advantages over their synthetic chemical counterparts. Virus capsids are a logical starting point for the development of new drug delivery systems, having evolved to protect and efficiently deliver their encapsulated genetic material into cells.^[^ ^18^ ^]^ Virus‐like particles (VLPs) are self‐assembling virus capsid proteins that lack the viral genome and naturally interact with polyanions, which enables electrostatic packaging of other guest molecules such as quantum dots, siRNA, and other proteins.^[^ ^19^, ^20^, ^21^ ^]^ Packaging can serve to both reduce the intrinsic toxicity of the guest molecule and protect the cargo from degradation by nucleases and proteases.^[^ ^22^, ^23^ ^]^ Non‐viral cage‐forming proteins have also been engineered to encapsulate a variety of cargoes,^[^ ^24^ ^]^ and directed evolution has shown that an icosahedral lumazine synthase can be adapted to package supercharged green fluorescent protein (GFP), enzymes, and nucleic acids.^[^ ^25^, ^26^, ^27^, ^28^ ^]^

Computational protein design methods have now repeatedly demonstrated atomic‐level control over the structures of custom‐designed self‐assembling proteins.^[^ ^29^, ^30^, ^31^, ^32^, ^33^ ^]^ These designed protein nanomaterials are often highly stable and can be modularly engineered to introduce new functions—two key features that often hamper VLP engineering efforts.^[^ ^34^ ^]^ Recent work has demonstrated that designed “one‐component” (i.e., homomeric) particles can be engineered to deliver siRNA to cells as effectively as lipofectamine but with less toxicity,^[^ ^35^ ^]^ and can encapsulate water‐insoluble small molecule drugs within a hydrophobic surfactant core.^[^ ^36^ ^]^ However, the static, pre‐assembled nature of these nanoparticles means that they are limited to passive cargo loading and release through large pores, limiting control over these important processes. These limitations can in principle be addressed by designing two‐component protein nanomaterials constructed from two distinct types of protein building blocks. The assembly of two‐component nanoparticles in vitro from independently purified components allows control over the assembly process itself and enables the encapsulation of cargos larger than their pores.^[^ ^32^, ^37^ ^]^ Here we investigate the versatility and robustness of cargo packaging and in vivo delivery using computationally designed two‐component protein nanoparticles.

## 2 Results

### 2.1 Controlled Encapsulation of Macromolecular Cargoes by In Vitro Assembly of I53‐50‐V5 Nanoparticles

**Figure 1:**
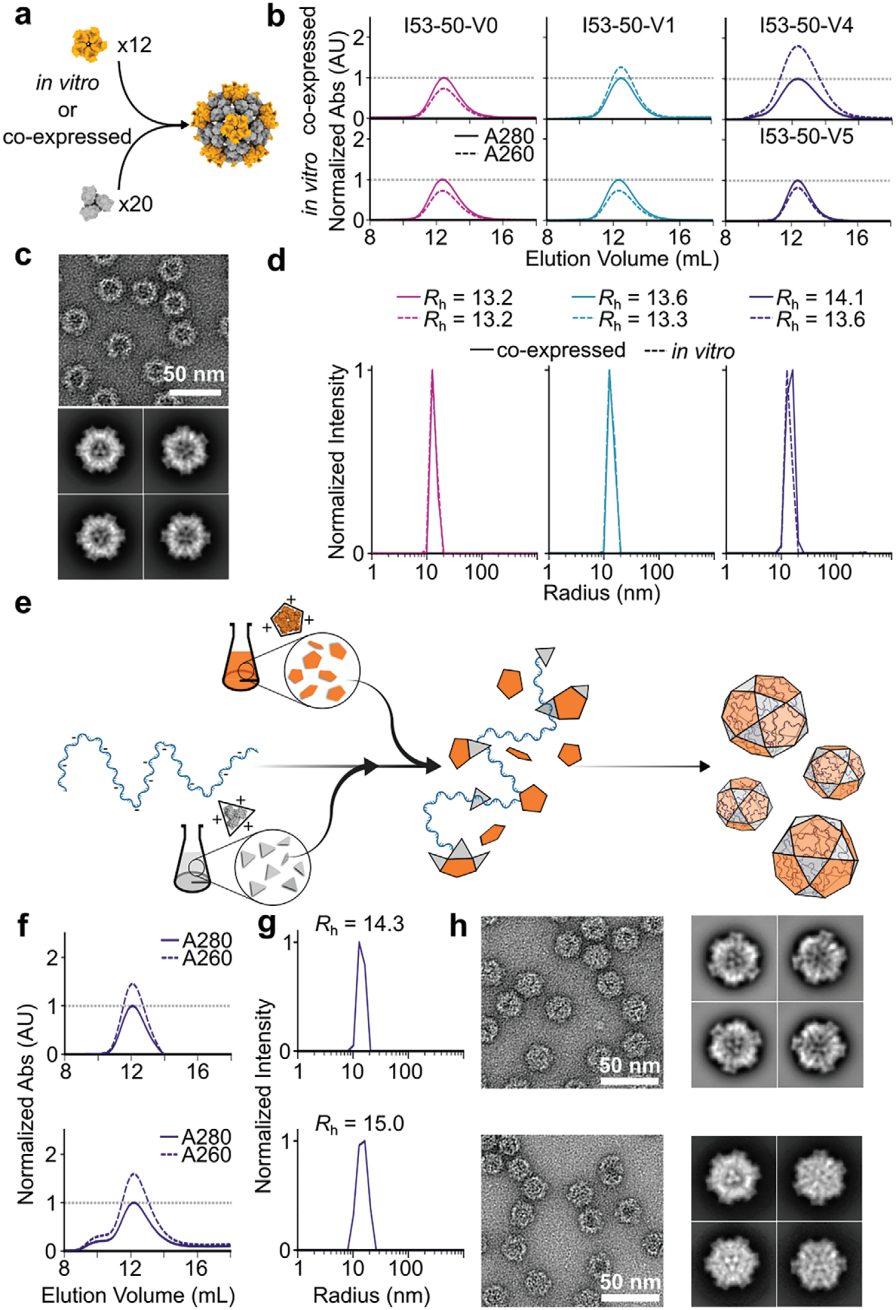
Controlled encapsulation of macromolecular cargoes by in vitro assembly of I53‐50‐V5 nanoparticles. a) Schematic of the computationally designed two‐component icosahedral protein nanoparticle I53‐50. Each nanoparticle consists of 12 copies of a pentameric building block (orange) and 20 copies of a trimeric building block (gray). These assemble spontaneously in *E. coli* cells when co‐expressed, or upon controlled mixing in vitro after independent expression and purification. b) SEC of I53‐50 variants produced by co‐expression in *E. coli* (top) or in vitro assembly (bottom). Absorbance was measured at 260 and 280 nm to detect the presence of packaged nucleic acid contaminants. Each chromatogram was normalized such that the peak absorbance at 280 nm equaled 1 for ease of comparison. Full chromatograms are provided in Figure S1d (Supporting Information). c) Representative negatively stained electron micrograph and 2D class averages of in vitro‐assembled I53‐50‐V5 particles. d) DLS measurements of the nanoparticles from panel (b) assembled by co‐expression in *E. coli* or by mixing in vitro assembly. Hydrodynamic radii (*R*_h_) are indicated. e) Schematic of electrostatic encapsulation of negatively charged cargoes via in vitro assembly. f–h) Biophysical characterization of I53‐50‐V5 assembled in the presence of two different sizes of nucleic acid cargoes, 400 nt (top) and 2500 nt (bottom), by (f) SEC, (g) DLS, and (h) nsEM (left, field view; right, selected 2D class averages).

We recently described I53‐50‐V4, a variant of a computationally designed two‐component icosahedral nanoparticle^[^ ^32^ ^]^ with a highly positively charged lumenal surface that was evolved to efficiently encapsulate and protect the mRNA encoding its two protein subunits in various environments, including for several hours in the circulatory system of mice.^[^ ^38^ ^]^ This genotype–phenotype linkage was achieved by mRNA packaging during nanoparticle production and assembly inside *Escherichia coli* cells, where each cell acts as a compartment that allows packaging of a specific mRNA. Although powerful, this approach cannot be used to controllably package other classes of cargoes, such as small molecules or non‐biological polymers. We reasoned that assembling I53‐50‐V4 in vitro could enable the generation of highly pure and monodisperse nanoparticles that efficiently package arbitrary molecular cargoes. Although we previously demonstrated encapsulation of a protein using a prototype version of this nanoparticle, I53‐50‐V1 (ref. [32]), we hypothesized that the improved packaging capabilities of I53‐50‐V4 obtained during its evolution would be reflected in superior cargo packaging in vitro. This approach requires the separate expression and purification of the trimeric and pentameric components of the nanoparticle (**Figure** **1**a). However, we found that the mutations introduced during the evolution of I53‐50‐V4 prevented soluble expression and purification of the pentameric component in the absence of the trimer. This inability to produce the I53‐50‐v4 pentamer necessitated the generation of variants that could be independently expressed and purified to enable in vitro assembly with the I53‐50‐V4A trimer. To identify the specific mutations responsible for destabilization of the I53‐50‐V4 pentamer, we computationally modeled a set of variants in which each evolved mutation was reverted to its original identity in the pentameric component of the precursor nanoparticle I53‐50‐V1, which can be purified independent of the trimer.^[^ ^32^ ^]^ The E24F mutation was identified as the most destabilizing, and we were able to purify soluble pentamer featuring a reversion to glutamic acid at this position in good yield (Figure S1, Supporting Information). We refer to the assembled nanoparticle containing this updated I53‐50B pentamer variant as I53‐50‐V5.

To confirm that I53‐50‐V5 still adopts the designed icosahedral architecture when assembled in vitro, we mixed equimolar amounts of the trimeric and pentameric subunits of I53‐50, I53‐50‐V1, and I53‐50‐V5 and compared the resulting nanoparticles. Size exclusion chromatography (SEC), dynamic light scattering (DLS), and negative stain electron microscopy (nsEM) indicated that all three assembly reactions generated monodisperse assemblies of the expected size (Figure 1b–d and Figure S1d, Supporting Information). Two‐dimensional classification of particles in negatively stained electron micrographs (Figure 1c and Figure S2a, Supporting Information) confirmed that I53‐50‐V5 adopted the known icosahedral morphology of I53‐50 (ref. [32]). The ratio of absorbance at 260:280 nm observed during SEC was <1 for all three nanoparticles, indicating that they did not contain substantial amounts of nucleic acid contaminants (Figure 1b and Figure S1d, Supporting Information). By contrast, co‐expressing the two subunits of I53‐50‐V4 in *E. coli* and purifying “pre‐assembled” nanoparticles from the soluble fraction of cell lysates resulted in a 260:280 nm ratio of 1.8 during SEC, confirming its known ability to package mRNA.^[^ ^38^ ^]^ Co‐expressed I53‐50‐V1 also packaged nucleic acid, although less efficiently, while the absorbance of co‐expressed I53‐50‐V0 indicated that it did not package substantial amounts of nucleic acid.

We then tested the ability of the evolved I53‐50‐V5 to encapsulate small (400 nt) and large (2500 nt) single‐stranded RNA (ssRNA) cargoes via in vitro assembly. In vitro encapsulation reactions were prepared by mixing the I53‐50‐V5A trimer with ssRNA prior to adding the I53‐50‐V5B pentamer to initiate assembly at a 1:1 molar ratio of cargo molecules to assembled nanoparticles (Figure 1e). This order of addition prevented aggregation of the pentamer prior to assembly, and we did not observe aggregation or changes in solubility in assembly reactions containing nucleic acid (Figure 1e–h). Purification of the reaction products by SEC showed that the fractions containing assembled nanoparticles had high absorbance at 260 nm relative to 280 nm, confirming that the ssRNA co‐eluted with the assembled nanoparticles, and we did not observe free nucleic acid (Figure 1f). Analysis of these nanoparticles by DLS and nsEM with two‐dimensional class averaging indicated that their size and morphology were not substantially altered by nucleic acid encapsulation (Figure 1g,h and Figure S2b–c, Supporting Information). Together, these data establish that in vitro assembly of I53‐50‐V5 from separately purified components generates pure, monodisperse icosahedral nanoparticles with minimal contaminating nucleic acid that can package defined cargoes through simple and controlled assembly in vitro.

### 2.2 Protection of Encapsulated ssRNA from Nuclease Challenge

**Figure 2:**
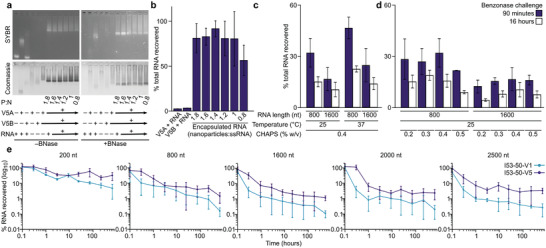
Protection of encapsulated ssRNA from nuclease challenge. a) Native agarose gel electrophoresis of encapsulation reactions containing increasing amounts of 400 nt ssRNA. P:N, protein:nucleic acid ratio (moles nanoparticle:ssRNA). b) RT‐qPCR quantitation of ssRNA recovery after Benzonase treatment of encapsulation reactions in panel (a). c) RT‐qPCR quantitation of ssRNA recovery after Benzonase treatment of 800 and 1600 nt ssRNA encapsulation reactions at 25 and 37 °C. The 25 °C data also appear in panel (d). d) RT‐qPCR quantitation of ssRNA recovery after Benzonase treatment of 800 and 1600 nt ssRNA encapsulation reactions at 25 °C and various CHAPS concentrations. e) Decay of encapsulated ssRNA of various lengths as function of the duration of Benzonase challenge. RT‐qPCR was used to quantify full‐length ssRNA present after incubation with Benzonase for 1 min, 20 min, 1 h, 3 h, 9 h, 24 h, 3 days, 9 days, and 27 days. In all panels, error bars indicate standard deviations from triplicate measurements.

We used ssRNA encapsulation and protection from nuclease challenge to explore the efficiency of cargo encapsulation in I53‐50‐V5 and its sensitivity to assembly conditions. Electrophoretic mobility shift assays (EMSA) indicated that while the I53‐50‐V5A trimer alone interacted with a 400 nt ssRNA, only encapsulation reactions containing both components provided robust protection from a 90 min challenge with excess Benzonase, indicating that assembly of the I53‐50‐V5 nanoparticle is required (**Figure** **2**a,b). Analysis of protected RNA extracted from I53‐50‐V5 nanoparticles after Benzonase challenge for 3 or 16 h confirmed the presence of full‐length 400 and 1600 nt ssRNA at both timepoints, although there was clear evidence of degradation of a fraction of the 1600 nt ssRNA (Figure S3a, Supporting Information). The migration profiles of encapsulation reactions containing 0.8–1.8 nanoparticles per 400 nt ssRNA exhibited no significant differences in mobility, and increasing the number of nanoparticles per ssRNA during encapsulation beyond 1:1 did not appear to increase the amount of ssRNA encapsulated and protected from Benzonase. RT‐qPCR of RNA recovered from these encapsulations confirmed this result and also revealed an apparent lower ssRNA recovery from the encapsulation reaction containing 0.8 nanoparticles per ssRNA, suggesting that the nanoparticles do not encapsulate multiple ssRNAs (Figure 2b). Together, these data indicate that assembly and encapsulation of 400 nt ssRNA was efficient and insensitive to slight alterations in the nanoparticle:ssRNA ratio above 1:1.

Current models of nucleic acid encapsulation by virus capsid proteins postulate capsid protein–protein interactions, capsid–cargo interactions, and cargo size as key parameters that control assembly and packaging.^[^ ^39^ ^]^ We encapsulated two different ssRNA cargoes (800 or 1600 nt) under a variety of experimental conditions intended to modulate these parameters. First, we modulated capsid–cargo interactions by varying ionic strength. We found that encapsulation was only possible at NaCl concentrations below 300 mm, presumably by allowing for sufficient electrostatic protein–cargo interactions (Figure S3b, Supporting Information). We then tested the effects of temperature and the concentration of the detergent CHAPS (3‐((3‐cholamidopropyl) dimethylammonio)‐1‐propanesulfonate), an excipient that enhances I53‐50‐V5B solubility, on ssRNA encapsulation and protection. We previously found that CHAPS can modulate the efficiency of in vitro assembly of our designed nanoparticles in a concentration‐dependent manner, presumably by competing with the designed hydrophobic interactions that drive assembly.^[^ ^37^ ^]^ We challenged ssRNA‐packaging nanoparticles, assembled at either 25 or 37 °C in the presence of 0.4% (v/v) CHAPS, with excess Benzonase for either 1.5 or 16 h (Figure 2c). We found that the packaged ssRNA trended toward being better protected in encapsulation reactions performed at 37 °C than the same reactions performed at room temperature. We also observed a trend toward better protection of the 800 nt ssRNA than the 1600 nt ssRNA at both temperatures: after 1.5 h of Benzonase challenge, the amount of full‐length 800 nt ssRNA recovered was more than twofold higher and reached ≈45% at 37 °C. We then measured protection in encapsulation reactions prepared at 37 °C in which the CHAPS concentration ranged from 0.2–0.5% (Figure 2d). Although the amounts of full‐length ssRNA recovered were variable, the data suggested that the maximum amount of encapsulated and protected ssRNA was obtained in the presence of 0.4% CHAPS. Although the differences we observed in these experiments were not statistically significant, the optimal encapsulation conditions we identified (150 mm NaCl, 37 °C, 0.4% CHAPS) consistently improved sample preparation across multiple in vitro assembly and encapsulation experiments.

Finally, we compared the ability of I53‐50‐V5 and I53‐50‐V1 to protect ssRNA molecules of various sizes from nuclease challenge. ssRNA molecules ranging from 200–2500 nt in length were encapsulated, subjected to a time course of nuclease challenge spanning from 1 min to 27 days at 25 °C, and extracted for amplification and quantification of remaining full‐length cargo by RT‐qPCR (Figure 2e). I53‐50‐V5 protected a substantially higher proportion of most ssRNA cargoes than I53‐50‐V1, even at early time points. The amount of full‐length ssRNA recovered from both nanoparticles decayed gradually over longer periods of nuclease challenge, with a trend toward more durable protection of shorter ssRNA cargoes. The smallest ssRNA (200 nt) exhibited the slowest rate of decay, as I53‐50‐V5 protected nearly all of the cargo from Benzonase for 10 min, 41% for 3 h, and 32% for 27 days. Protection of the larger ssRNA cargoes was less efficient overall; 1–10% of each full‐length ssRNA cargo was recovered from I53‐50‐V5 after 27 days of nuclease challenge, compared to less than 1% recovered from I53‐50‐V1. These data are consistent with analysis of protected 1600 nt RNA extracted from I53‐50‐V5 nanoparticles after Benzonase challenge for 3 or 16 h, which confirmed that although full‐length 1600 nt ssRNA was present at both timepoints, protection was less efficient for this RNA than for the shorter 400 nt ssRNA (Figure S3a, Supporting Information). Together, these results establish that two‐component nanoparticles can encapsulate and partially protect ssRNA cargoes under a variety of experimental conditions. They are also consistent with the previously noted improved packaging and protection afforded by I53‐50‐V5 compared to its I53‐50‐V1 precursor, as the evolved nanoparticle was approximately an order of magnitude more effective in protecting full‐length ssRNA cargoes. The improved protection acquired during evolution of I53‐50 was previously attributed to optimization of the number and locations of charged amino acids near the small pore at the icosahedral twofold axes.^[^ ^38^ ^]^

### 2.3 Encapsulation of Radiant Star Polymer Nanoparticles

**Figure 3:**
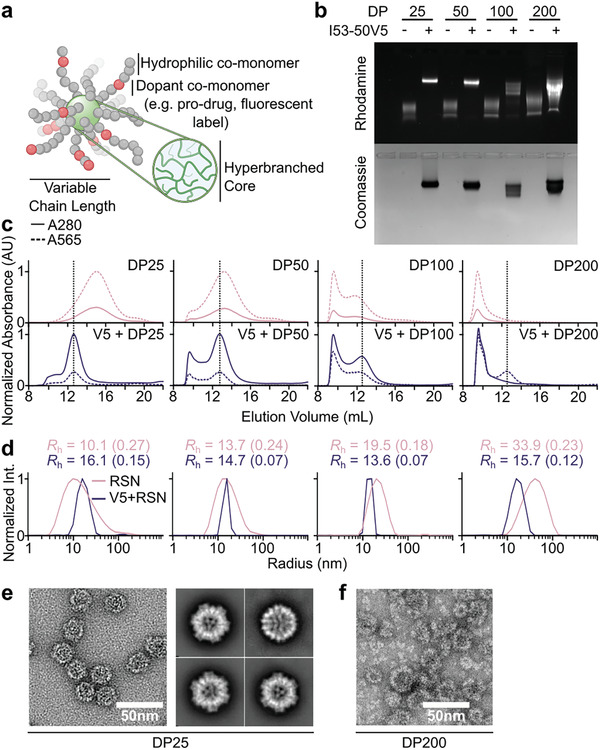
Encapsulation of non‐biological polymeric nanoparticles inside a designed protein nanoparticle. a) Cartoon representation of the structure of a radiant star polymer nanoparticle. b) Native agarose gel electrophoresis of increasing sizes of RSN present upon encapsulation. c) SEC trace of RSN alone (top) and RSN encapsulated within I53‐50‐V5 (bottom). Small polymers, once encapsulated, eluted at the same elution volume as empty I53‐50‐V5 (dotted vertical line). d) DLS size and polydispersity measurements of RSN alone or encapsulated within I53‐50‐V5. e) Representative negatively stained electron micrograph and class averages of DP25 encapsulated within I53‐50‐V5 particles. f) Negatively stained electron micrograph of a DP200 encapsulation reaction. The data shown are from representative experiments that were performed at least twice.

We further explored the ability of I53‐50‐V5 to controllably package various classes of molecules by synthesizing and encapsulating several different radiant star polymer nanoparticles (RSNs),^[^ ^40^ ^]^ a recently described class of polymer nanostructures in which linear chains are grown from a hyperbranched, hydrophilic core using reversible addition−fragmentation chain‐transfer (RAFT) polymerization (**Figure** **3**a). We reasoned that the compact, well‐defined three‐dimensional architectures of RSNs would enable more efficient packaging than linear polymers that would require compaction during packaging. To enable the electrostatic encapsulation approach described above for nucleic acids, RSNs were designed using an anionic monomer, mono‐2‐(methacryloyloxy)ethyl succinate (SMA), for the radiant arms. Each monomeric subunit of the resultant polymers thus carried a single negative charge. The length of the linear chains, and therefore the hydrodynamic radius of the polymer nanoparticle, was controlled by altering the degree of polymerization (DP) via the ratio of monomer to chain transfer agent (CTA) in the RAFT reaction. We targeted four different degrees of polymerization (DP25, DP50, DP100, and DP200) to study the ability of I53‐50‐V5 to encapsulate synthetic polymers of varying size. A small amount of rhodamine B methacrylate (RhMA) was co‐polymerized into the linear arms to enable detection of the polymers. After purification, molecular weights and hydrodynamic radii were established through aqueous gel permeation chromatography (SEC‐MALS) and DLS (Figure S4, Supporting Information) and indicated that the RSNs ranged from 225 kDa (*R*_h_ = 10.1 nm) to 1.7 MDa (*R*_h_ = 33.9 nm).

Following the conditions established above for nucleic acid packaging, the four RSNs were each encapsulated at a 1:1 nanoparticle:RSN ratio. Each RSN was resuspended in buffer and mixed with the I53‐50‐V5A trimer followed by the I53‐50‐V5B pentamer to allow assembly to proceed for an hour at 37 °C. We took advantage of the rhodamine incorporated into the RSNs and their polyanionic nature deriving from the negatively charged SMA monomer to visualize encapsulation by EMSA (Figure 3b). In the absence of I53‐50‐V5, the RSNs migrated rapidly through agarose gels, with the smaller polymers migrating furthest. All four encapsulation reactions yielded significantly shifted fluorescent RSN bands that were also stained by Coomassie, indicating co‐migration of I53‐50‐V5 and each RSN. The reactions for the two smallest RSNs, DP25 and DP50, both yielded single sharp bands, suggesting formation of a monodisperse species. By contrast, multiple smeared bands were observed after encapsulation of the larger RSNs, DP100 and DP200, suggesting inefficient encapsulation and the formation of multiple distinct species.

We further characterized RSN encapsulation by SEC, DLS, and nsEM. The water‐soluble nature of RSNs, and their similar size to assembled I53‐50, enabled the side by side comparison of free RSNs and encapsulation reactions by SEC. Chromatograms of free RSNs or encapsulation reactions were collected at 565 and 280 nm to track the rhodamine in the RSN and the protein nanoparticle, respectively (Figure 3c). While the two smallest RSNs are well resolved by SEC, a significant amount of DP100 and nearly all of DP200 elute at the void volume, consistent with their large size. When encapsulated within I53‐50‐V5, the 565 nm peaks of both DP25 and DP50 shift to precisely match the elution of monodisperse I53‐50‐V5 nanoparticles around 13 mL. Although a small amount of the DP25 encapsulation reaction, and slightly more of that of DP50, eluted at the void volume, no residual signal at 565 nm was observed at the unencapsulated elution volume, indicating that all polymer is efficiently encapsulated. Consistent with the EMSA, SEC suggested inefficient encapsulation of the two larger RSNs within I53‐50‐V5. Although a small rhodamine peak co‐eluted with I53‐50‐V5 in the case of DP100, the majority of the RSN in both encapsulation reactions eluted at the void volume. Interestingly, most of the protein also eluted at the void volume, suggesting that the larger polymers caused protein aggregation. DLS of SEC fractions corresponding to the elution peak near 13 mL indicated that monodisperse I53‐50‐V5 nanoparticles could be obtained after packaging each RSN, although in lower yield for the larger polymers (Figure 3d). DLS of the free RSNs exhibited relatively broad size distributions that increased with the degree of polymerization as expected. Analysis of the smallest (DP25) and largest (DP200) encapsulations by nsEM confirmed the biophysical characterization. In vitro encapsulation of DP25 resulted in monodisperse particles, with 2D class averages closely resembling those seen for empty I53‐50‐V5 and encapsulations of short ssRNA (Figure 3e and Figure S2d, Supporting Information). Meanwhile, we observed large aggregates and a substantial amount of unassembled protein in encapsulation reactions using DP200, consistent with the SEC and DLS data indicating that this polymer is too large to fit in the I53‐50‐V5 lumen (Figure 3f).

### 2.4 Synthesis of a Resiquimod Prodrug Monomer and Resiquimod‐Loaded RSN

Resiquimod is an antiviral imidazoquinoline that has been shown to activate the innate immune system via Toll‐Like Receptors (TLR) 7 and 8 (ref. [41]). While it has been approved for use in certain topical indications, it is underutilized as a vaccine adjuvant due to systemic toxicity and poor tolerability in humans.^[^ ^42^ ^]^ Like other small molecules, it is prone to diffuse away from the injection site, and there has been substantial interest in new formulation and delivery methods for resiquimod and other imidazoquinolines.^[^ ^43^, ^44^, ^45^, ^46^, ^47^ ^]^ The emergence of two‐component protein nanoparticles (including I53‐50) as a clinically relevant platform for nanoparticle vaccine development^[^ ^48^, ^49^, ^50^, ^51^ ^]^ motivated the encapsulation of resiquimod inside the I53‐50‐V5 nanoparticle. Previous studies have suggested that an encapsulation approach to formulation could improve safety and tolerability by directly co‐delivering adjuvant with antigen to antigen‐presenting cells while also potentially lowering the amount required for activity.^[^ ^47^, ^52^ ^]^ We incorporated resiquimod into an RSN polymer to enable straightforward in vitro encapsulation inside I53‐50‐V5 and subsequent release in vivo. Building polymeric prodrug architectures using polymerizable drug monomers provides a viable approach for incorporating multiple components such as drugs, targeting moieties, and labeling agents simultaneously without any laborious post‐synthetic procedures.^[^ ^40^, ^47^, ^53^, ^54^ ^]^

**Figure 4:**
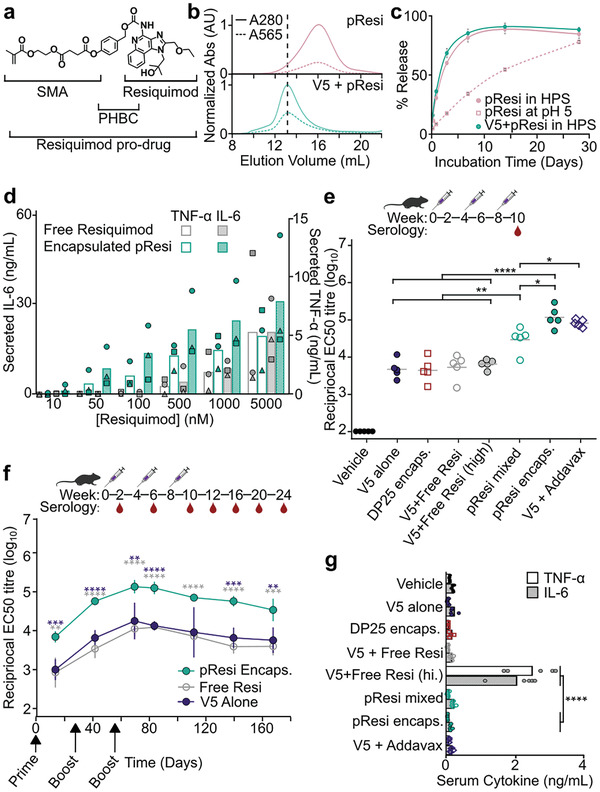
Encapsulated polymeric adjuvants improve immune responses in vitro and in vivo. a) Schematic of resiquimod prodrug monomer that is polymerized into RSNs via RAFT. b) SEC trace of pResi alone (top) and pResi encapsulated within I53‐50‐V5 (bottom). Once encapsulated, the polymer nanoparticle elutes earlier from the column, at the same elution volume as empty I53‐50‐V5, indicating it is now trapped within the protein nanoparticle. c) Kinetics of release of free resiquimod from pResi alone in human serum or buffer at pH 5 and encapsulated pResi in human pooled serum (HPS). d) In vitro cytokine response from human PBMCs (each of three donors represented by a distinct symbol) incubated for 24 h with free resiquimod or encapsulated pResi. e) Serum IgG responses against I53‐50‐V5 after three immunizations of BALB/c mice (*n* = 5), measured as EC_50_ titer. f) Anti‐I53‐50‐V5 antibody titers measured over 24 weeks for groups receiving encapsulated pResi (green), free resiquimod (gray), or no adjuvant (purple). g) In vivo systemic cytokine responses measured 1 h post‐immunization. Only free resiquimod at a dose commonly used in the literature induced TNF‐α and IL‐6 levels above background. The data shown are from representative experiments that were performed at least twice. Error bars in panel (f) indicate standard deviation. Statistical significance was determined by an independent *t*‐test with Bonferroni correction; ****, *p* ≤ 1 × 10^−4^; ***, *p* ≤ 1 × 10^−3^; **, *p* ≤ 1 × 10^−2^; *, *p* ≤ 5 × 10^−2^.

Additionally, by adjusting the molar ratio of the monomers, the loading of each component can be precisely controlled with a well‐defined statistical distribution that allows for maintenance of the polymers negative surface charge to facilitate encapsulation within two‐component protein nanoparticles. We designed a methacrylate‐based prodrug monomer carrying a *para*‐hydroxybenzyloxycarbonyl (PHBC) spacer between the polymerizable monomer (SMA) and the resiquimod (**Figure** **4**a) that can be directly polymerized by the RAFT method. The PHBC spacer is well known for its self‐immolative nature; once the phenolic ester bond is hydrolyzed, the resulting phenolate quickly self‐eliminates and triggers the spontaneous release of the intact drug.^[^ ^55^, ^56^ ^]^ The monomer synthesis starting from SMA is outlined in Scheme S1 (Supporting Information). The successful synthesis of resiquimod methacrylate (ResMA) monomer was confirmed by ^1^H NMR spectroscopy (Figure S5, Supporting Information) and ESI‐MS signal at *m*/*z* value of 677.4 [M+H]^+^.

Having identified an RSN size range that allows efficient encapsulation within I53‐50‐V5, a polymeric nanoparticle that incorporated the resiquimod prodrug monomer was then synthesized. The smallest RSN size (DP25) was targeted to ensure efficient encapsulation. Following co‐polymerization of ResMA, RhMA, and SMA to the poly‐4‐Cyano‐4‐((ethylsulfanylthiocarbonyl)sulfanyl)pentanoic acid hydroxyethyl methacrylate (pECT HEMA) core using [2,2′‐Azobis(4‐methoxy‐2,4‐dimethylvaleronitrile)] (V70) initiator, the purified polymerized resiquimod nanoparticle (pResi) yielded a 96% monomer conversion and a resiquimod drug weight percent of 9.74% (Figure S6, Supporting Information). Characterization of pResi by SEC‐MALS and DLS (Figure S4, Supporting Information) indicated a low dispersity (*Đ*) of 1.1, a hydrodynamic radius of 9.8 nm and a total molecular weight of 376 kDa, matching the size of the RSN observed to efficiently encapsulate above.

### 2.5 Encapsulation and Release of pResi In Vitro

pResi was encapsulated within I53‐50‐V5 using the same stoichiometric mixing and assembly approach described above for the RSNs. Encapsulation was confirmed by SEC, which showed a clear shift in elution volume of pResi (monitored by rhodamine absorbance at 565 nm) from 16 mL to the expected I53‐50‐V5 elution volume of 13 mL (Figure 4b). Very little residual unencapsulated polymer was observed, indicating efficient encapsulation by in vitro assembly. Assuming a single polymer was encapsulated per nanoparticle and the molecular weights and drug loading above, we estimate ≈100 molecules of resiquimod were packaged per I53‐50‐V5 nanoparticle.

We studied the rate at which resiquimod is released from pResi via cleavage of the PHBC linker. Figure 4c shows the amount of resiquimod released at 37 °C as a function of time from pResi alone or encapsulated in I53‐50‐V5 when incubated in human serum or buffer at pH 5 to simulate the acidic conditions within the endosome. Encapsulated pResi could not be studied at pH 5 due to the tendency of I53‐50‐V5 to flocculate at low pH. No difference was observed in the rate of release of resiquimod in human serum regardless of encapsulation state (*t*_1/2_ of 1.5 d vs 1.9 d for encapsulated vs non‐encapsulated pRESI), suggesting that the presence of the protein nanoparticle might not impact drug release in circulation. At low pH, the release rate of resiquimod from pResi was significantly slower (*t*_1/2_ = 19.6 d), suggesting that drug release from the polymer architecture may slow after endocytosis of the encapsulated nanoparticle.

### 2.6 Encapsulated pResi Provides Potent Immune Activation without Systemic Toxicity

After confirming that resiquimod was released from the polymer prodrug via hydrolysis, we tested the ability of the encapsulated pResi to induce cytokine production by peripheral blood mononuclear cells (PBMCs) in vitro. Human PBMCs from three donors were incubated for 24 h with varying concentrations of free resiquimod or pResi encapsulated within I53‐50‐V5. Compared with dose‐matched free resiquimod, encapsulated pResi appeared to elicit higher levels of secreted Interleukin‐6 (IL‐6) and Tumor Necrosis Factor‐alpha (TNF‐α) across the concentration series until saturating concentrations were reached (5 µm), although this difference did not reach statistical significance (Figure 4d). Empty I53‐50‐V5 and pResi alone each resulted in much lower activation (Figure S7, Supporting Information), suggesting that the pResi is more readily taken up by cells when encapsulated and that empty I53‐50‐V5 has a minimal impact on immune stimulation.

Having demonstrated the biological activity of encapsulated pResi in vitro, we then evaluated its effects in immunization studies in mice. We used I53‐50‐V5 itself as the immunizing antigen and measured anti‐I53‐50‐V5 binding titers after three immunizations with a series of distinct formulations. In addition to encapsulated pResi containing 0.36 µg of resiquimod, we tested pResi mixed with pre‐assembled I53‐50‐V5 (i.e., non‐encapsulated), I53‐50‐V5 formulated with 0.36 or 20 µg of free resiquimod, I53‐50‐V5 encapsulating the DP25 RSN lacking resiquimod, I53‐50‐V5 alone, and I53‐50‐V5 formulated with AddaVax, a squalene‐based oil‐in‐water emulsion we have used as an adjuvant in previous immunization studies.^[^ ^48^, ^49^, ^50^ ^]^ The amounts of I53‐50‐V5 and resiquimod administered were matched in all relevant groups except for the high dose of free resiquimod (20 µg), which was selected based on typical doses used in previous studies in mice.^[^ ^57^ ^]^ Formulating I53‐50‐V5 with free resiquimod at either dose resulted in no improvement over the encapsulated DP25 RSN or I53‐50‐V5 alone (Figure 4e). In contrast, encapsulated pResi elicited serum antibody titers against I53‐50‐V5 that were significantly higher than non‐encapsulated pResi and were comparable to AddaVax. Plotting the kinetics of the serological response, which was followed for four months following the second boost, showed that encapsulated pResi elicited a durable response in which the levels of antibodies against I53‐50‐V5 remained consistently higher than the groups receiving free resiquimod or no adjuvant (Figure 4f).

To determine the effect of polymerizing resiquimod and packaging it into I53‐50‐V5 on systemic toxicity, we measured serum cytokine levels 1 h after immunization, a commonly used timepoint to evaluate toxicity.^[^ ^44^ ^]^ TNF‐α and IL‐6 were elevated when 20 µg of free resiquimod was delivered (Figure 4g). By contrast, levels of these cytokines were indistinguishable from background in all other groups, including pResi encapsulated in I53‐50‐V5, which elicited antibody levels that were significantly higher than all other formulations except AddaVax (Figure 4e). These data indicate that encapsulating resiquimod in I53‐50‐V5 significantly increased immunogenicity without causing the systemic cytokine secretion that has been associated with toxicity in previous studies of this adjuvant.^[^ ^43^ ^]^

## 3 Discussion

Here we have shown that computationally designed two‐component protein nanoparticles can efficiently package and protect multiple macromolecular cargoes via in vitro assembly. Although many previous studies have demonstrated cargo packaging inside self‐assembling protein nanoparticles,^[^ ^58^, ^59^ ^]^ notably VLPs, this study establishes that custom nanoparticles that have been predictively designed to have specific structural features^[^ ^32^ ^]^ and subsequently engineered to enable cargo packaging^[^ ^38^ ^]^ can be assembled around various cargoes in vitro using purified solutions of defined components. This approach as a whole, comprising predictive nanoparticle design through in vitro cargo encapsulation, offers an unprecedented level of control over the structure, function, and manufacturing of cargo‐packaging protein nanoparticles, which should facilitate practical application of this promising class of nanotechnologies.

Our data show that I53‐50‐V5 nanoparticles encapsulating either ssRNA or non‐biological polymers retain their predicted structure and are highly monodisperse, showcasing the modularity with which designed two‐component nanoparticles can be engineered. Our EM data establish that I53‐50‐V5 adopts the same computationally designed structure as the original I53‐50 nanoparticle, despite bearing 25 amino acid substitutions across the two subunits. We note that I53‐50 and several other two‐component nanoparticles also maintain their morphologies when displaying many different proteins on their exteriors as genetic fusions.^[^ ^48^, ^50^, ^60^, ^61^ ^]^ Together, these observations establish that the interior and exterior surfaces of computationally designed two‐component nanoparticles can be modularly engineered for various functions without altering their structures. This is advantageous because it enables stepwise design of nanoparticle structure and function.

Our data also demonstrate that electrostatic encapsulation via in vitro assembly of two‐component nanoparticles is a versatile approach to cargo packaging. We highlight two aspects in particular. First, in vitro assembly can be used to package a wide variety of macromolecular cargoes. We previously packaged protein guests using the I53‐50‐V0 precursor to I53‐50‐V5 (ref. [32]), and here have used the evolved variant to more efficiently package both nucleic acids and synthetic polymers incorporating small molecule drugs. We showed that encapsulation with I53‐50‐V5 has cargo‐specific advantages depending on the material encapsulated. The nucleic acid is protected against degradation, and the polymeric prodrug is protected against esterase enzymes that might cleave the resiquimod more rapidly compared to hydrolytic controlled release. The co‐localization of antigen with resiquimod also may allow more efficient co‐delivery to antigen presenting cells (APCs) and improve vaccination efficacy. The ability to package such divergent cargoes is a direct consequence of the simple mechanism of encapsulation: all three components (two nanoparticle components and a cargo) are mixed in vitro at stoichiometric ratios, and packaging occurs during nanoparticle assembly due to non‐specific electrostatic interactions between the cargo and the lumenal surfaces of the nanoparticle components. These interactions are maintained irrespective of the specific sequence of the nucleic acid or the chemical nature of the polyanionic backbone. We note that VLPs, which have evolved cationic interior surfaces in order to package their genomes during assembly in vivo, can be similarly repurposed to package a wide variety of polyanionic cargoes in vitro.^[^ ^62^ ^]^ Second, in vitro assembly provides a simple mechanism for encapsulating defined cargoes by compartmentalization in reaction chambers (e.g., test tubes), resulting in pure and monodisperse cargo‐bearing nanoparticles. In this approach, the two‐component nature of I53‐50‐V5 is advantageous in that the trimeric and pentameric components can each be purified to homogeneity prior to assembly via simple mixing in the presence of a defined cargo, enabling versatile and controllable encapsulation.

Although our goal was not to match the packaging efficiency of viruses, it is interesting to compare the performance of I53‐50‐V5 to them. While I53‐50‐V5 efficiently packaged 200–400 nt ssRNA and polymeric nanoparticles ≈350 kDa in molecular weight, we found that the efficiency of encapsulation and the quality of the resultant nanoparticles was poorer for larger cargoes. At 23 nm in diameter, I53‐50‐V5 is approximately the same size as small, simple bacteriophages like MS2 and Qβ. However, these viruses encapsulate ssRNA genomes that are substantially larger (3.6 and 4.2 kb, respectively^[^ ^63^ ^]^) than the largest ssRNA we encapsulated (2.5 kb). This highly efficient packaging results from the co‐evolution of genomic material and capsid protein, leading to, among other features, the presence of “packaging signals” in the nucleic acid which compact it and interact with the capsid interior to enable selective packaging of the viral genome within a host cell.^[^ ^63^ ^]^ The emergence of packaging signals after extensive laboratory evolution of a non‐viral protein compartment that packages its own encoding RNA with an efficiency rivaling viruses^[^ ^28^ ^]^ suggests that the packaging efficiency of I53‐50‐V5 could be further improved with additional design or evolution.

We demonstrated the functional potential of cargo‐bearing I53‐50‐V5 nanoparticles in vivo by encapsulating and delivering a small molecule TLR7/8 agonist in immunization experiments in mice. We showed that a radiant star polymer was an ideal encapsulated material due to its statistical incorporation of functional monomers that maintain its negative surface charge and allow for high drug loading without the need for further compaction prior to encapsulation. While fully synthetic polymer encapsulation by VLPs has been explored previously, to our knowledge, this is the first time a fully synthetic polymer prodrug has been encapsulated.^[^ ^64^, ^65^, ^66^, ^67^ ^]^ Resiquimod‐packaged nanoparticles elicited more potent humoral immune responses against I53‐50‐V5 than I53‐50‐V5 mixed with a 55‐fold higher dose of free resiquimod, while simultaneously minimizing systemic toxicity. These results support several previous studies demonstrating that chemical linkage of resiquimod to an antigen improves B and T cell responses and outcomes after challenge,^[^ ^46^, ^47^, ^52^, ^68^ ^]^ and are consistent with the notion that the ideal vaccine formulation links adjuvant to antigen, thereby restricting immune stimulation to relevant lymphoid tissues and enhancing both B and T cell‐mediated protection.^[^ ^69^ ^]^

For the sake of simplicity in our proof of principle vaccine study, we used the I53‐50‐V5 nanoparticle itself as the antigen in addition to being the delivery vehicle. The potent antibody responses we observed suggest that immune responses against protein nanoparticles may be a significant obstacle to their use in many targeted delivery applications. Although recent clinical successes using viral vectors for gene therapy and vaccine delivery have established that protein‐based delivery systems can be effective in humans,^[^ ^70^ ^]^ vector‐neutralizing antibodies are a known issue, especially in the context of repeat administration.^[^ ^71^ ^]^ However, we suggest that anti‐nanoparticle antibodies may be less problematic in vaccine applications. We note that protein nanoparticle vaccines are distinct from viral vectors in that their cargoes are functional immediately upon inoculation (rather than requiring delivery and expression of a transgene), and several studies have suggested that antibodies against underlying nanoparticle scaffolds do not deleteriously affect responses against antigens displayed on their exteriors.^[^ ^48^, ^72^, ^73^, ^74^ ^]^ The robust immune responses we observed here highlight the potent immunogenicity of nanoparticle immunogens, and indeed multiple I53‐50‐based vaccines are currently being evaluated in clinical trials, with a SARS‐CoV‐2 vaccine based on I53‐50 recently being licensed for use in humans.^[^ ^48^, ^49^, ^51^ ^]^

There are several limitations to this study that can be addressed in future work. First, there are several other classes of cargoes with therapeutic potential that could benefit from being packaged in protein nanoparticles, such as Cas9 ribonucleoprotein complexes^[^ ^75^ ^]^ and small molecule cytotoxic drugs,^[^ ^16^ ^]^ although efficiently delivering such cargoes in vivo would require substantial further engineering to overcome additional challenges with in vivo delivery (e.g., endosomal escape). Second, we have not demonstrated delivery to specific cell types through the display of targeting domains on the protein nanoparticle exteriors, although the tolerance of two‐component nanoparticles to genetic fusion in principle makes this possible.^[^ ^48^, ^50^, ^60^ ^]^ Finally, future studies should consider the effect of immune responses against the nanoparticle on delivery to non‐lymphoid tissues; as mentioned above, this is known to be a challenge for gene delivery using viral vectors.^[^ ^71^ ^]^ With additional investigation, the sophisticated structural and functional properties of proteins and our growing ability to predictively design them^[^ ^76^ ^]^ may provide solutions to these and other challenges related to targeted delivery.

In conclusion, our demonstration that various cargoes of therapeutic relevance can be efficiently packaged via in vitro assembly extends the functional potential of computationally designed two‐component nanoparticles. Combined with the ability to predictively design their structures with atomic‐level accuracy and display a variety of functional domains via genetic fusion, our results position two‐component nanoparticles as a promising platform for drug delivery that could combine the strengths of protein biologics with those of other therapeutic modalities such as nucleic acids, small molecules, and non‐biological polymers.

## 4 Experimental Section

### Materials

Materials were purchased from Sigma Aldrich unless otherwise specified. Resiquimod was purchased from AstaTech. 4‐Cyano‐4‐(ethylsulfanylthiocarbonyl) sulfanylpentanoic acid (ECT) was obtained from Omm Scientific. [2,2′‐Azobis(4‐methoxy‐2,4‐dimethylvaleronitrile)] (V‐70) was purchased from FUJIFILM Wako Pure Chemical Corporation and used without further purification. Spectra/Por regenerated cellulose dialysis membranes were purchased from Spectrum Laboratories.

### Plasmid Construction

Gene sequences encoding the individual nanoparticle subunits of I53‐50‐V4 were amplified off of the bicistronic gene described in Butterfield et al.^[^ ^38^ ^]^ and cloned into pET29b+ (Novagen) using the NdeI and XhoI restriction sites. The F24E mutation and additional tags such as C‐terminal StrepTags were inserted through PCR and Gibson assembly. Amino acid sequences for all proteins used in this study are provided in Table S1 (Supporting Information).

### Protein Expression and Purification

Plasmids were transformed into T7 Express *E. coli* (New England BioLabs) and expressed overnight at 18 °C after cultures were induced with 1 mm Isopropyl β‐D‐1‐thiogalactopyranoside (IPTG). Expression cultures were pelleted by centrifugation and resuspended in lysis buffer composed of 50 mm Tris pH 8.0, 1 m NaCl, 0.75% (v/v) CHAPS, 20 mm Imidazole, 1 mm DTT, 1 mm PMSF, 0.1 mg mL^−1^ DNase, and 0.05 mg mL^−1^ RNase. The resuspended pellets were lysed by sonication and centrifuged to yield clarified supernatants containing His‐tagged protein of interest. These proteins were purified by immobilized metal affinity chromatography (IMAC) with a HisTrap High Performance column (Cytiva) after binding in lysis buffer as described above and eluted in a buffer containing 50 mm Tris pH 8.0, 1 m NaCl, 0.75% CHAPS, 500 mm Imidazole, and 1 mm DTT. After molecular weight confirmation by SDS‐PAGE, IMAC fractions containing the desired proteins were further purified by SEC using a Superdex S200 Increase 10/300 GL column (Cytiva) in a buffer containing 50 mm Tris pH 8.0, 1 m NaCl, 0.75% CHAPS and stored at −20 °C.

### In Vitro Assembly of I53‐50‐V5, ssRNA Encapsulation, and Nuclease Challenge

Prior to assembly, trimeric and pentameric nanoparticle components were separately diluted from storage buffer to concentrations ranging from 5 to 50 µm in 20 mm Tris pH 8.0, 150 mm NaCl, 0.375% CHAPS. For encapsulation reactions, molecular cargos (i.e., RNA or polymers) were added to the solution containing the trimeric component, and the assembly reactions were initiated by mixing the trimer/cargo‐ and pentamer‐containing solutions followed by overnight incubation at 37 °C unless otherwise indicated. 500 units of Benzonase (excess enzyme; enough to degrade ≈20 mg of RNA in 30 min), was added to the encapsulation reactions and incubated for the indicated times at room temperature. Tubes were tightly sealed and parafilmed to avoid sample evaporation. For samples that were intended for RNA protection quantification by RT‐qPCR, reactions were performed in triplicate.

### RNA Extraction and Quantification

After Benzonase challenge, RNA was extracted from encapsulation reactions using TRIzol (Thermofisher Scientific) and the Qiagen RNeasy kit according to the manufacturers’ instructions. Specifically, 100 µL of sample containing encapsulated RNA was added to 500 µL TRIzol, then 100 µL chloroform was added and the solution was mixed vigorously. After centrifugation for 10 min at 20 000 rcf, 200 µL of the aqueous phase was mixed with 200 µL of 100% ethanol and applied to an RNeasy spin column for purification according to the manufacturer's instructions, and eluted with a 1:100 solution of RNaseOUT recombinant RNase Inhibitor (Thermofisher Scientific) diluted into nuclease‐free water.

Extracted RNA was reverse transcribed using Primescript reverse transcriptase (Takara Bio) for 1 h at 52 °C with a primer universal to all of the RNAs used in this study (5′‐CAAACAACAGATGGCTGGCAC‐3′). Thus, a 10 µL reaction contained: 1 µL dNTPs (10 mm each), 1 µL DTT (100 µm), 1 µL Primescript reverse transcriptase, 2 µL cDNA synthesis buffer, 1 µL RNaseOUT, 1 µL primer (10 µm), 2 µL purified RNA template, and 1 µL nuclease‐free dH_2_O. 2 uL of each sample was then analyzed by qPCR using reactions containing 5 µL Kapa HiFi HotStart Readymix (Roche), 0.5 µL SYBR green (diluted 1:20 in nuclease‐free water), 0.5 µL of each primer (10 µm), and 1.5 µL nuclease free water. Triplicate standard curves consisting of three tenfold dilutions were prepared for each RNA length and verified to be linear upon quantification.

### RNA Synthesis

RNA was synthesized via in vitro transcription using HiScribe T7 High Yield RNA Synthesis Kit (New England BioLabs) following the manufacturer's instructions. After the synthesis was completed, reactions were incubated in DNase to remove template DNA. RNA transcripts were purified using RNAclean XP beads (Beckman Coulter Life Sciences).

### Dynamic Light Scattering

Dynamic light scattering was performed on a DynaPro NanoStar (Wyatt Technology Corp.). Measurements were performed in triplicate in a 1 µL quartz cuvette. Each measurement collected 10 acquisitions at 5 s per acquisition.

### Negative Stain Electron Microscopy

Negative stain electron micrographs were collected on a Talos model L120C electron microscope (Thermo Scientific). Protein nanoparticles were diluted to a concentration of 0.1 mg mL^−1^ in 25 mm Tris pH 8, 500 mm NaCl. Sample was applied to carbon coated 300 mesh copper grids (Ted Pella) which had been glow discharged immediately before use. The grids were washed and then stained with 0.75% (w/v) uranyl formate stain, immediately blotted, and then stained again.

### Electrophoretic Mobility Shift Analysis

A 2% (w/v) agarose gel was prepared by dissolving agarose (Fisher Scientific) in TAE buffer. SYBR Gold Nucleic Acid Gel Stain (Invitrogen) was added to the gel before casting. Electrophoresis was performed at 100 V for 1 h. After imaging nucleic acid, gels were incubated overnight in Gelcode Blue Stain Reagent (Thermo Scientific) for imaging of protein bands.

### Monomer Synthesis

Synthetic Scheme S1 (Supporting Information) was followed to obtain the Resiquimod prodrug (ResMA) monomer (**6**). Rhodamine B methacrylate (RhMA) was synthesized and fully characterized as described previously.^[^ ^53^ ^]^ All synthesized compounds were purified by precipitation and/or silica gel column chromatography techniques. The successful synthesis and purity of the monomers were confirmed and characterized by ^1^H NMR spectroscopy (Bruker Avance Spectrometer 300 MHz) and Electrospray Ionization‐Mass spectrometry (Bruker Esquire ion trap mass spectrometer). In brief, intermediate **4** was synthesized following previously reported methods.^[^ ^77^ ^]^ To **4** (1.26 g, 2.93 mmol) in 10 mL dichloromethane at 0 °C, was added methyl trifluoromethanesulfonate (0.31 mL, 2.83 mmol) in 2 mL dichloromethane dropwise over 5 min. After 10 min at 0 °C, the reaction mixture was stirred at room temperature for 1.5 h and then concentrated to 4 mL under reduced pressure. This solution was precipitated into diethyl ether in 50 mL conical centrifuge tubes (40 mL ether per tube), vortexed and centrifuged to isolate the product as a sticky pellet. This pellet was again treated with diethyl ether (40 mL per tube), vortexed, and centrifuged to get the intermediate **5**. This intermediate was dissolved in 15 mL CH_2_Cl_2_ and added to Resiquimod (692 mg, 2.20 mmol) in 35 mL CH_2_Cl_2_ at room temperature. The resulting reaction mixture was stirred for 21 h and then the solvent was rotary evaporated under reduced pressure. The crude product was purified by silica gel column chromatography using 3% methanol in chloroform to afford resiquimod prodrug monomer ResMA (**6)**. The reaction yielded 1.14 g, a 76.5% conversion. ^1^H NMR (300 MHz, DMSO‐d_6_) given in Figure S5 (Supporting Information) shows expected product formation: δ 1.13 (t, *J* = 6.9 Hz, 3H) 1.18 (s, 6H), 1.85 (s, 3H), 2.70 (t, *J* = 6.3 Hz, 2H), 2.85 (t, *J* = 6.3 Hz, 2H), δ 3.53 (q, *J* = 6.9 Hz, 2H), δ 4.31 (s, 4H), δ 4.55–45.12 (2 s and 1 brs merged, 5H), 5.21 (s, 2H), 5.66 (s, 1H), 6.02 (s, 1H), 7.11 (d, *J* = 8.7 Hz, 2H), 7.45–7.58 (1 d and 1 t merged, 3H), 7.63 (t, *J* = 7.5 Hz, 1H), 7.94 (d, *J* = 7.8 Hz, 1H), 8.54 (d, *J* = 8.1 Hz, 1H), 9.94 (s, 1H). MS (ESI, *m*/*z*): calculated for C_35_H_40_N_4_O_10_ (M): 676.3, found: 677.4 [M+H]^+^.

### pResi Core Synthesis Poly(HEMA‐ECT) (pHECT)

Synthesis of the macro‐CTA (pHECT) was described previously.^[^ ^40^ ^]^ The RSN core was synthesized by RAFT homopolymerization of the HEMA‐ECT in DMSO‐d_6_ under a nitrogen atmosphere using ABCVA (V501) as the radical initiator. The Degree of Polymerization (DP) of HEMA‐ECT was 10 and the initiator was 10% of the HEMA‐ECT. HEMA‐ECT (500 mg, 1.33 mmol), ABCVA (V501) (37.3 mg, 0.133 mmol), 1,3,5‐trioxane (7.4 mg), and DMSO‐d_6_ (1.286 g) were added in a 5 mL round bottom flask. The flask was sealed and the reaction mixture was degassed by bubbling nitrogen into the solution for 35 min and was then placed in a preheated oil bath at 70 °C for 18 h. The reaction was stopped by introducing oxygen by removing the septum and cooling the solution with a mild stream of air. The crude polymer was isolated by repeated precipitations into diethyl ether (8 times). Acetone was used to dissolve the polymer in between precipitations. The purified and dried polymer was characterized by ^1^H NMR spectroscopy. *T*_initial_ and *T*_final_^1^H NMR spectra of the crude solution indicated 99% monomer conversion, using an internal standard (1,3,5‐trioxane) added into the polymerization solution. After purification of unreacted monomers, the polymer was characterized by ^1^H NMR spectroscopy in CDCl_3_. All peaks characteristic of poly(HEMA‐ECT) were observed.

### RSN Synthesis

Copolymerization of SMA and RhMA was conducted in DMSO in the presence of pHECT (macro‐CTA) and ABCVA (initiator, I) following a synthetic procedure outlined previously.^[^ ^40^ ^]^

### pResi Synthesis

Copolymerization of SMA, ResMA, and RhMA was conducted in DMSO in the presence of poly(hECT) (macro‐CTA) and V‐70 (initiator, I). The initial molar feed percentages of each methacrylate monomer were 91.3, 7.8, and 0.9 mol%, respectively. The [M]_0_ : [CTA]_0_ : [I]_0_ was 25 : 1 : 0.05 at an initial monomer concentration of 94.5 wt%. To a 5 mL round bottom flask was added SMA (200 mg, 0.869 mmol), ResMA (50 mg, 0.074 mmol), RhMA (5 mg, 0.008 mmol), poly(hECT) (14.2 mg, 0.0377 mmol), and V70 (0.581 mg, 0.002 mmol) in a 2 mL total volume of DMSO. The solution was septa sealed and purged with nitrogen for 30 min. It was then transferred to a preheated oil bath at 30 °C and allowed to polymerize for 18 h. After the solution cooled, the polymer was purified by ether precipitation. The polymer was added dropwise to a 50 mL conical tube containing 45 mL diethyl ether and vigorously vortexed, followed by centrifugation at 3750 rpm, 4 °C for 5 min. The ether supernatant was decanted, and the polymer pellet was air dried and resolublized in 1 mL DMSO. This process was repeated for a total of 3 ether precipitations, followed by one ether wash.

To determine the degree of polymerization of monomer, 20 µL of the reaction mixture was taken before (*T*_initial_) and after (*T*_final_) the 18 h polymerization reaction, mixed with 700 µL DMSO‐d_6_, and analyzed via ^1^H NMR spectroscopy. The total molar fraction of monomer converted to polymer was calculated by comparing the vinyl resonances (3H) between 5.0 and 6.5 ppm from the two spectra. Following purification, ^1^H NMR spectra were recorded in the presence of fasudil hydrochloride (eNovation Chemicals) internal standard to determine the Resiquimod drug weight percent of the RSN (Figure S6, Supporting Information).

### Size Exclusion Chromatography (SEC‐MALS)

The average molecular mass (Mn) and dispersity of the RSNs were determined using 100% mass recovery and multiangle light scattering (MALS) SEC. The running solvent was 25 mm Tris‐HCl pH 8 with 150 mm NaCl (flow rate: 0.5 mL min^−1^) and samples were prepared at 5 mg mL^−1^. Separation was performed on a Phenomenex PolySep GFC‐P 6000 and data were collected by a Wyatt miniDAWN Treos and Wyatt Optilab rex. 100% mass recovery was performed using Wyatt's protocol with the refractive index data obtained from the Optilab rex. A dn/dc value of 0.164 resulted in accurate calculations of the injected mass across all RSNs characterized by SEC‐MALS. The dn/dc value was corroborated by performing a batch dn/dc determination over a range of RSN concentrations from 0.1–2 mg mL^−1^. Wyatt ASTRA software was used for data analysis and determination of absolute molecular weight and dispersity.

### pResi Encapsulation

Prior to encapsulation into I53‐50‐V5, pResi was dissolved into 25 mm Tris, pH 8 containing 0.3% CHAPS to a concentration of 10 mg mL^−1^. Assemblies were performed as described above at a stoichiometric ratio such that there were equimolar amounts of pResi and assembled I53‐50‐V5 nanoparticles in solution. The reaction mixture was incubated for 30 min at 37 °C before purification over a Superose 6 HR column (Cytiva) with a mobile phase of 25 mm Tris pH 8, 150 mm NaCl.

### Drug Release Assay

HPLC analysis of Resiquimod was performed using an Agilent 1260 HPLC equipped with Agilent ChemStation software (Palo Alto, CA, USA). The UV detector was operated at 254 nm. A Zorbax SB‐C18 analytical column (2.1 × 100 mm, 3.5 µm; Agilent Technologies, CA, USA) was used at ambient temperature with mobile phases of 1% acetic acid in acetonitrile and aqueous 1% (v/v) acetic acid with 5% (v/v) acetonitrile. With a 15 µL sample injection volume, the gradient method involved an increase from 2–95% (v/v) organic phase over the first 24 min at 0.2 mL min^−1^, 95–100% (v/v) organic from 24–25 min, and then a ramp down from 100–2% organic from 26.5–37 min at 0.35 mL min^−1^.

pResi RSNs either suspended in 1:1 PBS:human serum or 10 mm sodium phosphate pH 5, 150 mm NaCl; or encapsulated in I53‐50‐V5 nanoparticles in 1:1 PBS:serum, were incubated at 37 °C at a concentration of 100 µm resiquimod in a final volume of 100 µL for 28 days. At prescribed time points (0 h, 3 h, 8 h, 1 d, 3 d, 7 d, 14 d, 28 d), samples were transferred to −80 °C until all samples could be processed together. Resiquimod was extracted from each sample by 2:1 acetonitrile extraction at 4 °C and centrifugation at 18 000 rcf, 4 °C for 20 min. Supernatants containing resiquimod were filtered through a Millex GV low protein binding PVDF filter (0.22 µm; Millipore, Burlington, MA, USA) and nitrogen evaporated (Biotage, Uppsala, Sweden) for 90 min at 13 psi. Samples were resuspended in 100 µL deionized water and run against a resiquimod standard curve.

### Immunogen Preparation

Nanoparticles encapsulating pResi were prepared as described above. For groups receiving I53‐50‐V5 with free resiquimod or mixed with pResi, assemblies were prepared by mixing I53‐50‐V5A and I53‐50‐V5B in equimolar amounts. Assembly reactions were incubated at 37 °C for 30 min before an additional molar equivalent of I53‐50‐V5A was added to the reaction and allowed to sit at 4 °C overnight before purification on a Superose 6 10/300 HR column using a buffer of 25 mm Tris pH 8, 150 mm NaCl. This additional equivalent of I53‐50‐V5A was added to ensure that nanoparticles were completely formed, with no missing trimeric components.^[^ ^37^ ^]^ Following purification, nanoparticles were mixed with free resiquimod (prepared from a stock solution in DMSO) or pResi which had been dissolved into the same buffer.

### Endotoxin Measurement and Removal

Endotoxin was removed from nanoparticle components during protein purification using a detergent wash during IMAC. Proteins were immobilized on a 5 mL HisTrap HP column (GE Healthcare) equilibrated with buffer (25 mm Tris pH 8, 500 mm NaCl, 0.75% CHAPS) and the column was washed with ≈10 CV of the equilibration buffer. Elution was performed with a linear gradient of 0 to 500 mm imidazole in equilibration buffer. Fractions containing the desired protein were pooled and stored in a buffer containing detergent until assembly. Purified proteins were tested for endotoxin prior to assembly using a Charles River EndoSafe PTS system, and measured concentrations were routinely below 100 EU mL^−1^. Detergent was removed from assembled nanoparticles prior to immunization by buffer exchange via SEC into 25 mm Tris pH 8, 1500 mm NaCl.

### Mice

Female C57BL/6J mice were sourced at the age of 4 weeks from Jackson Laboratory, Bar Harbor, Maine (stock #000664). Animals were maintained at the Comparative Medicine Facility at the University of Washington, Seattle, WA, which is accredited by the American Association for the Accreditation of Laboratory Animal Care International (AAALAC). Animal procedures were performed with the approval and under the guidance of the Institutional Animal Care and Use Committee of the University of Washington, Seattle, WA under protocol number 4470‐01.

### Immunizations and Serum Collection

6‐week old mice (*n* = 5 per group) were immunized with 25 µg immunogen at week 0 and subsequently boosted at weeks 4 and 8. Mice were immunized by intramuscular injection at the quadriceps muscle using a 27‐gauge needle (BD, San Diego, CA) with 50 µL immunogen solution per leg (100 µL total) under isoflurane anesthesia. Blood samples were obtained via submental venipuncture using a 5 mm lancet (Braintree Scientific, Braintree, MA) 2 weeks after the initial immunization and every 2–4 weeks thereafter. For measurements of systemic cytokines, blood samples were obtained as above, 1 h post‐immunization. At the endpoint of week 12, mice were anesthetized with isoflurane for blood collection via cardiac puncture. The in vivo study was repeated twice. For measurement of response duration, blood samples were collected from the initial cohort of mice every 4 weeks for 24 weeks, before being anesthetized with isoflurane for terminal blood collection via cardiac puncture. Blood was rested in 1.5 mL Eppendorf tubes for 30 min at room temperature to allow for coagulation. Serum was then isolated from cellular components and clotting factors via centrifugation at 2000 g for 10 min. Serum was stored at −80 °C until use.

### Cell Culture

Frozen human PBMCs were thawed and washed in 40 mL PBS containing 10% (w/v) heat‐inactivated FBS, then 10 mL of complete RPMI 1640 (25 mm HEPES, sodium bicarbonate, 1 mm sodium pyruvate, 1 mm L‐glutamine, 1% (w/v) penicillin/streptomycin and 10% (w/v) FBS). Cells were counted and resuspended in complete RPMI 1640 at 2 × 10^6^ cells mL^−1^ and 0.25 mL per well was plated in 96‐well tissue culture plates. Resiquimod and its prodrug form were dissolved in DMSO and diluted in TBS to the desired concentration, maintaining a final concentration of DMSO in each well of less than 0.1% (v/v). pResi was dissolved into TBS and diluted in each well to the desired concentration. Encapsulated pResi was prepared as above and adjusted to the correct concentration using a standard curve based on the absorbance of rhodamine at 565 nm. After 24 h of incubation at 37 °C and 8% CO_2_, plates were centrifuged at 1500 rpm for 5 min and 0.2 mL of supernatants were removed and transferred to fresh 96‐well plates. Samples were stored at −80 °C before being analyzed by ELISA.

### Enzyme‐Linked Immunosorbent Assay

Enzyme‐linked immunosorbent assays (ELISA) were used to determine the levels of I53‐50‐V5‐specific antibodies in mouse sera. Maxisorp (Nunc) ELISA plates were coated overnight at 4 °C with 0.08 µg mL^−1^ of I53‐50‐V5 per well in 0.1 m sodium bicarbonate buffer, pH 9.4. Plates were then blocked with a 4% (w/v) solution of dried milk powder (BioRad) in TBS with 0.05% (v/v) Tween 20 (TBST) for 1 h at room temperature. Serial dilutions of sera were added to the plates and, after washing, antibody binding was measured using a hydrogen peroxidase‐coupled horse anti‐mouse IgG antibody. Plates were then washed thoroughly in TBST, colorimetric substrate (TMB, Thermo Fisher) was added, and absorbance was read at 450 nm. To quantify the presence of cytokines in cell culture and mouse sera, cytokine quantification kits were purchased from R&D Systems and used following the manufacturer's recommendations.

### Statistical Analysis

Graphs were plotted and statistical analysis was performed with code written in Python3 using the matplotlib and statannot packages. Statistical significance was determined by an independent *t*‐test with Bonferroni correction; ****, *p* ≤ 1 × 10^−4^; ***, *p* ≤ 1 × 10^−3^; **, *p* ≤ 1 × 10^−2^; *, *p* ≤ 5 × 10^−2^. Data were normalized when mentioned in the text or figure legends. Relevant sample size and error bar information is mentioned in the matching figure legends.

## Conflict of Interest

N.P.K. is a co‐founder, shareholder, paid consultant, and chair of the scientific advisory board of Icosavax, Inc. The King lab has received unrelated sponsored research agreements from Pfizer and GSK.

## Author Contributions

K‐L.H., I.S., D.E., A.W., A.J.C., P.S., and N.P.K. designed the study. K‐L.H., I.S., C.L.L., M.N.P., S.S., J.N., K.S.M., D.R., and A.N.P. performed experiments. K‐L.H., I.S., C.L.L, S.S., and N.P.K. wrote the manuscript. All authors analyzed data, discussed results, and commented on the manuscript.

## Supporting information

Supporting Information

## Data Availability

The data that support the findings of this study are available from the corresponding author upon reasonable request.
