# Supplementary material for: Macromolecular Cargo Encapsulation via In Vitro Assembly of Two‐Component Protein Nanoparticles
Source: Adv Healthc Mater. 2024 Feb 11;13(11):2303910. doi: 10.1002/adhm.202303910 (PMC11468305; doi:10.1002/adhm.202303910)
Supplement: Supplementary file 1 — Supporting Information [file ADHM-13-2303910-s001.pdf]

ADVANCED HEALTHCARE MATERIALS

Supporting Information

for *Adv. Healthcare Mater.*, DOI 10.1002/adhm.202303910

Macromolecular Cargo Encapsulation via In Vitro Assembly of Two-Component Protein Nanoparticles

Karla-Luise Herpoldt, Ciana L. López, Isaac Sappington, Minh N. Pham, Selvi Srinivasan, Jason Netland, Katherine S. Montgomery, Debashish Roy, Alexander N. Prossnitz, Daniel Ellis, Adam J. Wargacki, Marion Pepper, Anthony J. Convertine, Patrick S. Stayton* and Neil P. King**

Macromolecular cargo encapsulation via *in vitro* assembly of two-component protein nanoparticles

SUPPLEMENTARY INFORMATION

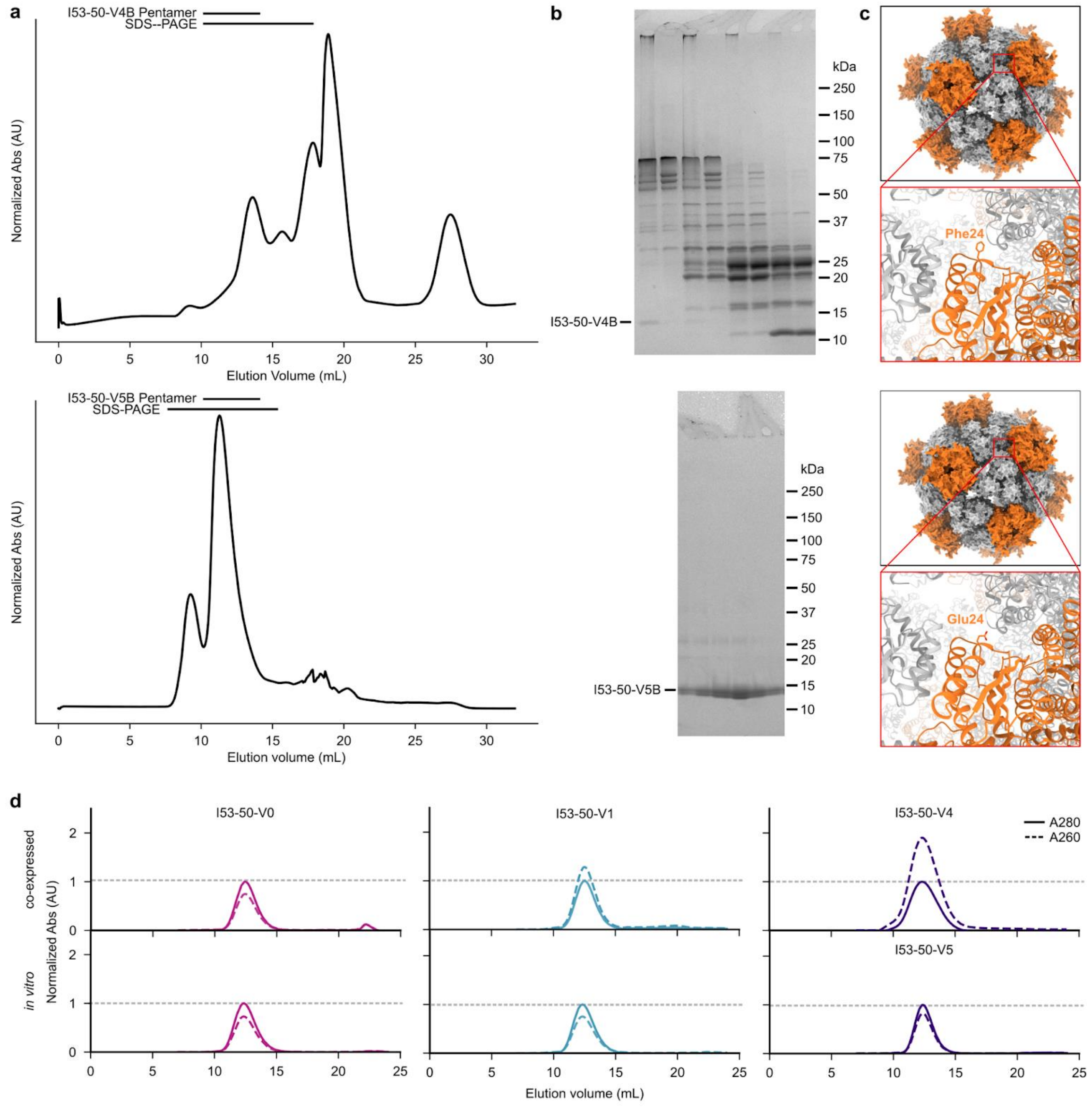

Supplementary Figure 1 | F24E mutation improves expression and purification of the I53-50-V5B pentamer relative to the I53-50-V4B pentamer. a, Chromatograms of preparative SEC runs of I53-50-V4B (top) and I53-50-V5B (bottom) using a Superdex 200 10/300 column. The fractions containing pentamer and analyzed by SDS-PAGE are indicated. **b,** SDS-PAGE of SEC fractions. The band corresponding to each pentamer is indicated. Purification of the I53-50-V4B pentamer yielded impure preparations containing little pentamer, while purification of the I53-50-V5B pentamer resulted in pure protein in good yield. **c,** Design models of I53-50-V4 (top) and I53-50-V5 (bottom). The F24E mutation is located in the pores of the assembled nanoparticle. **d,** Uncropped versions of the SEC chromatograms shown in **Fig. 1b**. All chromatograms on the bottom row correspond to unpurified *in vitro* assembly reactions.

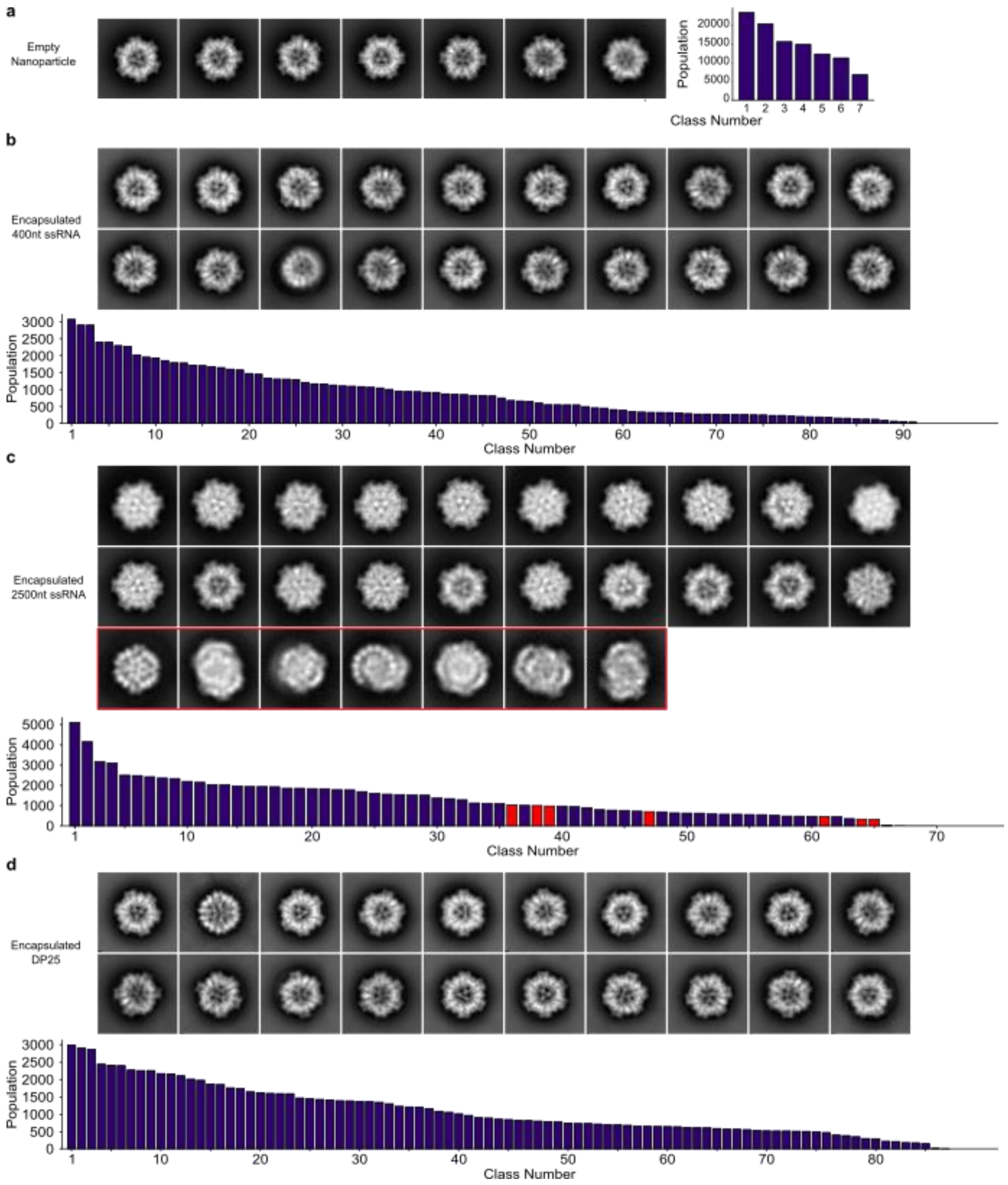

Supplementary Figure 2 | nsEM two-dimensional class averages of various nanoparticles. Selected 2D class averages of (**a**) empty I53-50-V5, (**b**) I53-50-V5 encapsulating 400 nt ssRNA, (**c**) I53-50-V5 encapsulating 2500 nt ssRNA, and (**d**) I53-50-V5 encapsulating DP25. For each nanoparticle, the twenty most populated classes are shown along with any observed aberrant classes (red). The histograms show the number of images in all 2D class averages for each nanoparticle, demonstrating that only a small fraction of the total nanoparticles encapsulating 2500 nt ssRNA adopt aberrant structures.

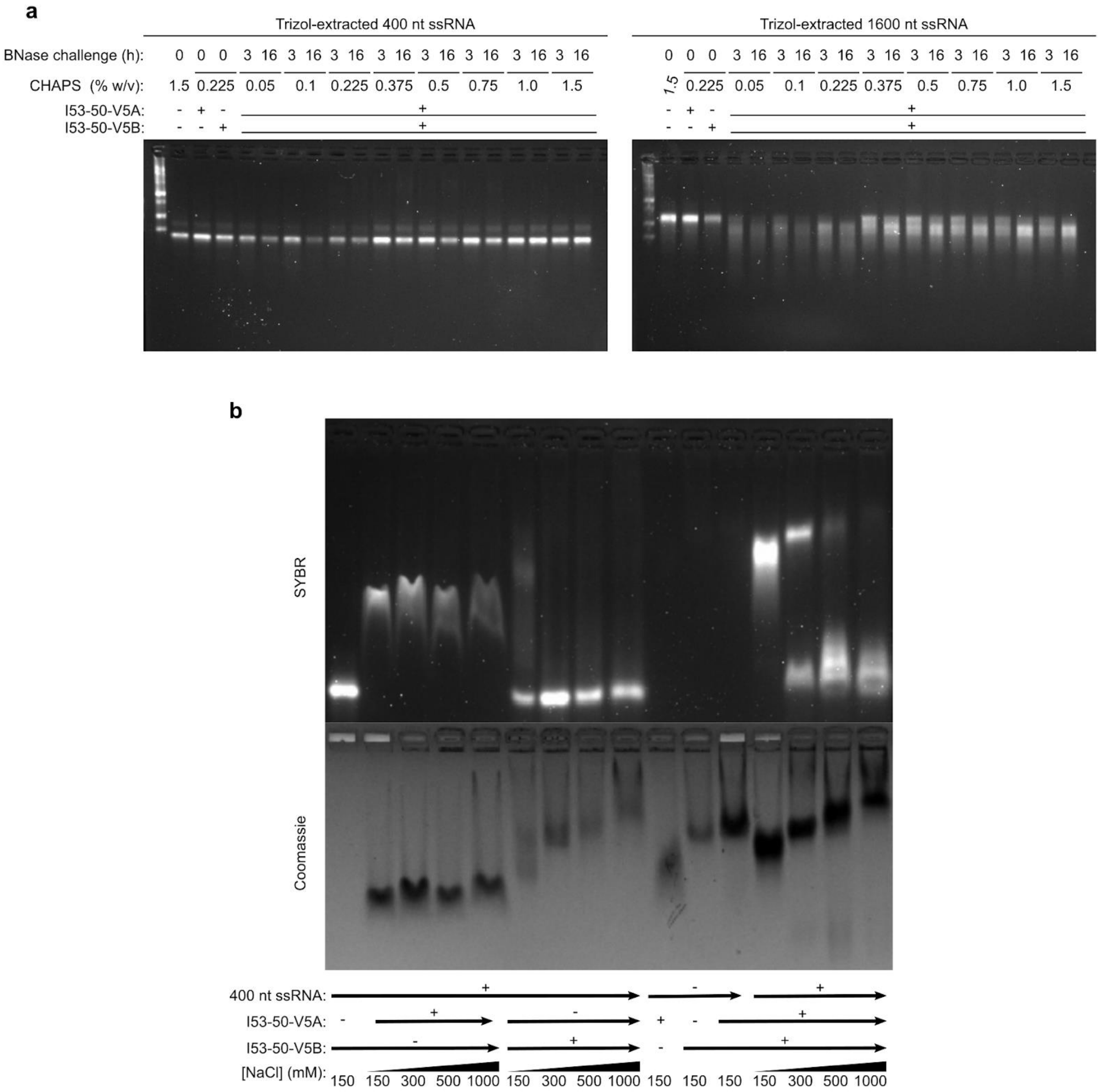

Supplementary Figure 3 | Recovery of full-length ssRNA after benzonase challenge and salt-dependent encapsulation efficiency of ssRNA by I53-50-V5. a, 400 nt and 1600 nt ssRNA was encapsulated, challenged with benzonase, extracted with TRIzol, and analyzed by agarose gel electrophoresis. After overnight benzonase challenge (500 U), full-length RNA was recovered in various amounts that depended on ssRNA length and detergent concentration. **b,** Native agarose gel electrophoresis of ssRNA, nanoparticle components, and encapsulation reactions. ssRNA encapsulation was most efficient at 150 mM NaCl. At higher [NaCl], ssRNA comigrates with trimeric I53-50-V5A but does not interact with pentameric I53-50-V5B.

a

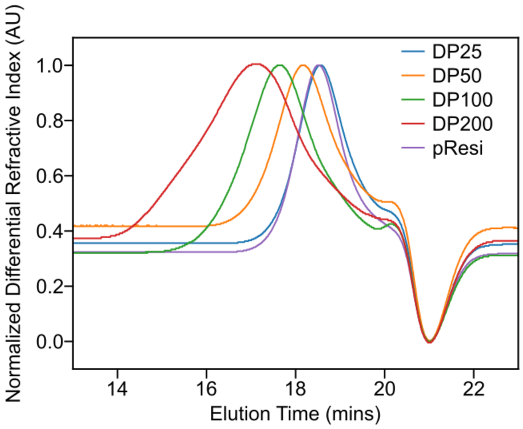

b

	**Mn (Da)**	**Đ**	**Rh (nm)**
DP25	225,900	1.13	10.1
DP50	348,600	1.25	13.7
DP100	670,000	1.45	19.5
DP200	1,720,000	1.72	33.9
pResi	376,000	1.11	9.8

Supplementary Figure 4 | Biophysical characterization of RSNs. a, Aqueous SEC-MALS of RSNs, showing dispersity of synthesized polymer nanoparticles. **b,** Quantification of average molecular mass by SEC-MALS, dispersity (Đ), and hydrodynamic radius by DLS.

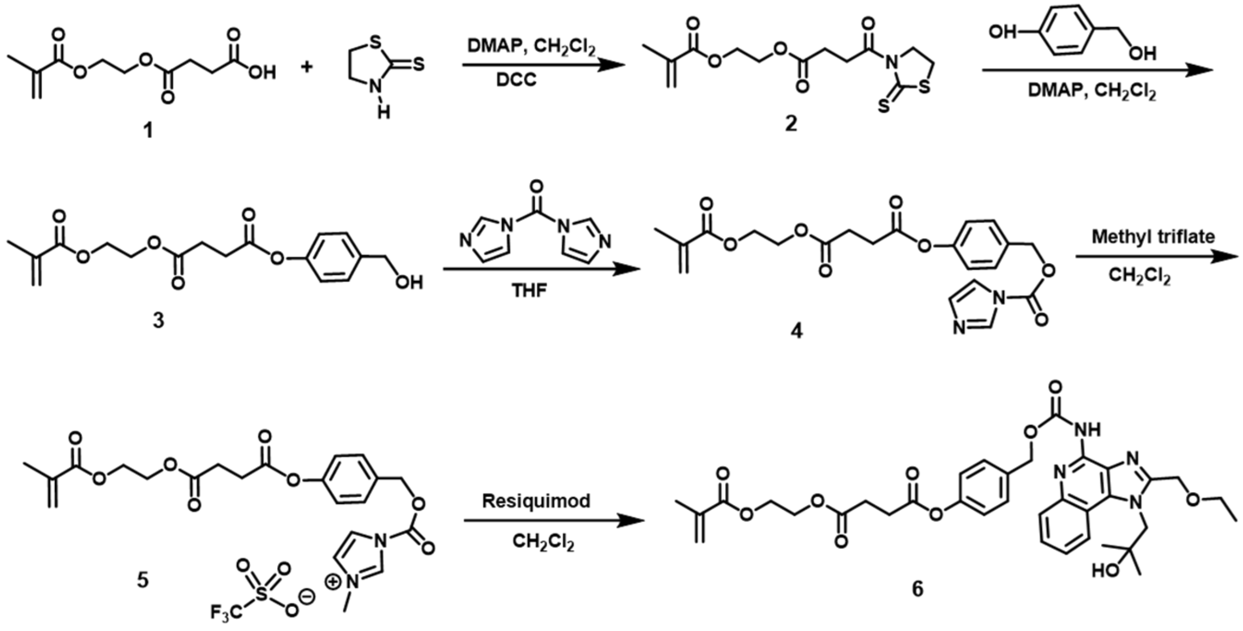

Scheme 1 | Synthesis of Resiquimod prodrug (ResMA) monomer 6.

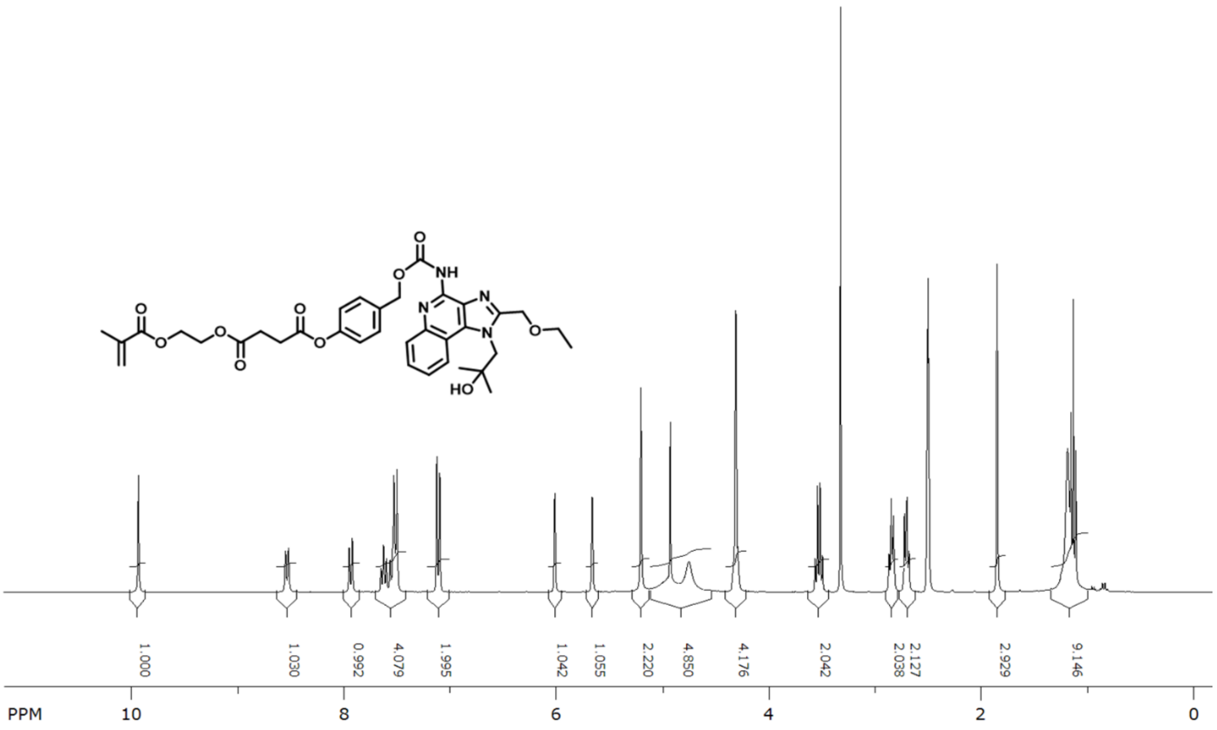

Supplementary Figure 5 | ^{1}H NMR spectrum of Resiquimod prodrug monomer ResMA (6) in DMSO-d_6.

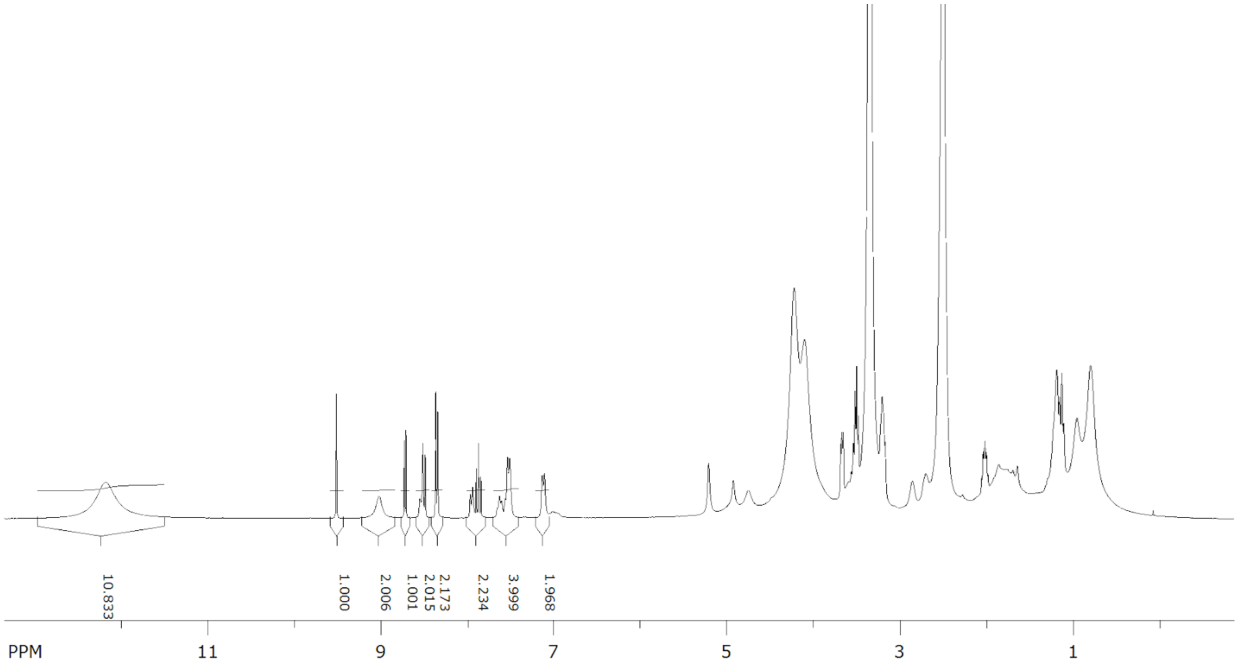

Supplementary Figure 6 | ^{1}H NMR spectrum of Resiquimod RSN with fasudil hydrochloride as internal standard in DMSO-d$_6$. Resiquimod drug weight % on the polymer was calculated via ^{1}H NMR spectroscopy using fasudil hydrochloride as the internal standard. NMR sample contained pResi 20 mg/mL and fasudil hydrochloride 2 mg/mL in DMSO-d$_6$. The ratio of integration of the resiquimod proton at 8.54 ppm to the fasudil proton at 8.72 ppm provided the resiquimod weight % as 9.72%.

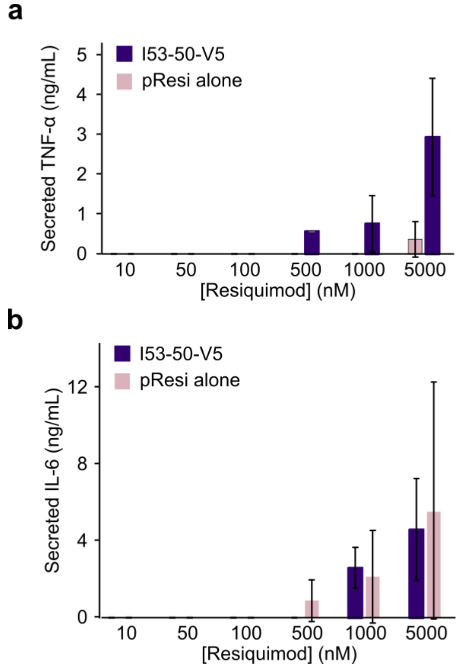

Supplementary Figure 7 | Cytokine levels measured from human PBMCs for empty I53-50-V5 and pResi nanoparticles. Levels of secreted TNF-alpha (**a**) and IL-6 (**b**) were measured from human PBMCs incubated with varying amounts of pResi alone (concentrations matched to free resiquimod in **Fig. 4d**) or I53-50-V5 alone. Although no resiquimod is present in the empty I53-50-V5 samples, the protein concentration used matches that from the corresponding encapsulated pResi samples in **Fig. 4d** to control for any stimulation resulting from the nanoparticle itself.

Table S1 | Amino acid sequences of proteins used in this study.

Protein	Amino Acid Sequence*	Notes
I53-50-V0A	MKMEELFKKHKIVAVLRANSVEEAIEKAVAVFAGGVHLIEITFT<br>VPDADTVIKALSVLKEKGAIIGAGTVTSVEQCRKAVESGAEFIV<br>SPHLDEEISQFCKEKGVFYMPGVMTPTELVKAMKLGHTILKL<br>FPGEVVGPQFVKAMKGPFPNVKFVPTGGVNLDNVCEWFKA<br>GVLAVGVGSALVKGTPDEVREKAKAFVEKIRGCTEHHHHHH	Original I53-50A trimer<br>(Bale et al., 2016)
I53-50-V1A†<br>(I53-50-A.1PT1)	MKMEELFKKHKIVAVLRANSVEEAIEKAVAVFAGGVHLIEITFT<br>VPDADTVIKALSVLKEKGAIIGAGTVTSVEQCRKAVESGAEFIV<br>SPHLDEEISQFCKEKGVFYMPGVMTPTELVKAMKLGHDILKL<br>FPGEVVGPQFVKAMKGPFPNVKFVPTGGVNLDNVCKWFKA<br>GVLAVGVGKALVKGKPDEVREKAKKFVKKIRGCTEGSLEHH<br>HHHH	Engineered I53-50A trimer<br>(Bale et al., 2016)
I53-50-V5A‡<br>(I53-50-V4A)	MGHHHHHHGGMTMEELFKRHTIVAVLRANSVEEAIEKAVAV<br>FAGGVHLIEITFTVPDADTVIKALSVLKEDGAIIGAGTVTSVDQ<br>CRKAVESGAEFIVSPHLDEEISQFCKEKGVFYMPGVMTPTEL<br>VKAMKLGHDILKLFPGEVVGPQFVKAMKGPFPNVKFVPTGG<br>VNLDNVCKWFKAGVLAVGVGNALVKGNPDKVREKAKKFVKK<br>IRGCTEGSWSHPQFEK	Evolved I53-50A trimer<br>(Butterfield et al., 2017)
I53-50-V0B	MNQHSHKDYETVRIAVVRARWHAEIVDACVSAFEAAMADIG<br>GDRFAVDVFDVPGAYEIPLHARTLAETGRYGAVLGTAFVVNG<br>GIYRHEFVASAVIDGMMNVQLSTGVPVLSAVLTPHRYRDSDA<br>HTLLFLALFAVKGMEAARACVEILAAREKIAAGSLEHHHHHH	Original I53-50B pentamer<br>(Bale et al., 2016)
I53-50-V1B†	MNQHSHKDHETVRIAVVRARWHAEIVDACVSAFEAAMRDIG<br>GDRFAVDVFDVPGAYEIPLHARTLAETGRYGAVLGTAFVVNG<br>GIYRHEFVASAVIDGMMNVQLDTGVPVLSAVLTPHNYDKSKA<br>HTLLFLALFAVKGMEAARACVEILAAREKIAAGSLEHHHHHH	Engineered I53-50B<br>pentamer<br>(Butterfield et al., 2017)
I53-50-V5B‡	MGSSHHHHHHSSGENLYFQGNQHSQKDQETVRIAVVRARW<br>HAEIVDACVSAFEAAMRKIGGERFAVDVFDVPGAYEIPLHAR<br>TLAKTGRYGAVLGTAFVVNGGIYRHEFVASAVIDGMMNVQLD<br>TGVPVLSAVLTPHNYDKSNAKTLLFLALFAVKGMEAARACVEI<br>LAAREKIAAGS	This study

*Purification tags and linkers are underlined.
†Differences in V1 constructs compared to V0 constructs are highlighted in yellow.
‡Differences in V5 constructs compared to V1 constructs are highlighted in green. The F24E mutation in I53-50-V5B relative to I53-50-V4B [38] is highlighted in orange.
